# Surface Potential Driven Water Harvesting from Fog

**DOI:** 10.1021/acsnano.1c01437

**Published:** 2021-04-26

**Authors:** Daniel P. Ura, Joanna Knapczyk-Korczak, Piotr K. Szewczyk, Ewa A. Sroczyk, Tommaso Busolo, Mateusz M. Marzec, Andrzej Bernasik, Sohini Kar-Narayan, Urszula Stachewicz

**Affiliations:** † Faculty of Metals Engineering and Industrial Computer Science, AGH University of Science and Technology, 30-059 Kraków, Poland; ‡ Department of Materials Science and Metallurgy, University of Cambridge, CB3 0FS Cambridge, United Kingdom; § Academic Centre for Materials and Nanotechnology, AGH University of Science and Technology, 30-059 Kraków, Poland; ∥ Faculty of Physics and Applied Computer Science, AGH University of Science and Technology, 30-059 Kraków, Poland

**Keywords:** electrospinning, electrical polarity, fog collectors, polycarbonate, surface potential, water harvesting

## Abstract

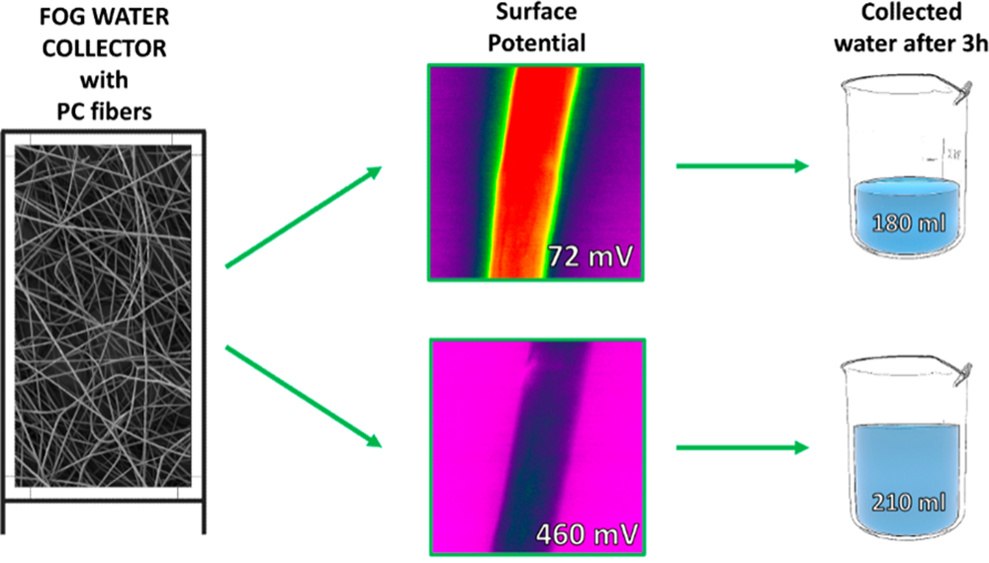

Access to clean water is a global challenge, and fog collectors are a promising solution. Polycarbonate (PC) fibers have been used in fog collectors but with limited efficiency. In this study, we show that controlling voltage polarity and humidity during the electrospinning of PC fibers improves their surface properties for water collection capability. We experimentally measured the effect of both the surface morphology and the chemistry of PC fiber on their surface potential and mechanical properties in relation to the water collection efficiency from fog. PC fibers produced at high humidity and with negative voltage polarity show a superior water collection rate combined with the highest tensile strength. We proved that electric potential on surface and morphology are crucial, as often designed by nature, for enhancing the water collection capabilities *via* the single-step production of fibers without any postprocessing needs.

In spite of the fact that the quality of life has significantly improved in the last century, 30% of the world’s population still struggles for access to drinking water. It is estimated that by 2025 two-thirds of the world population will have no access to clean drinking water.^1^ This global challenge requires innovative and sustainable solutions for the improvement of water-harvesting technologies. Fog is the perfect source of water, and Nature has learned to extract it in order to survive.^2^ One of the ways to imitate these systems is fog water collectors (FWCs).

Conventional FWCs are polymer (polypropylene (PP) and polyethylene (PE)) meshes mounted onto metal stands perpendicular to the fog-laden wind,^3^ whose efficiency, however, has room for improvement.^4^ The efficiency of FWCs is highly dependent not only on the applied materials but also on environmental conditions, including fog composition, wind velocity, and air humidity.^5^ The size of single FWC mesh ranges from several to several dozen square meters, wherein the ratio of width to height is not bigger than 2.5–3.^6^ The standard FWC can collect from 3 to 75 L·m^–2^ per day.^7^ The permeability of the mesh is the crucial parameter, which has significant influence on the water harvesting efficiency. Water droplets are captured by the mesh and form even larger clusters, which can block the fog flow and significantly inhibit the further water collection.^8^ The mesh geometry is essential for the efficient drainage in the water extraction process because the droplets run down to the special container thanks to gravity.^9^ The most popular FWCs consist of a double layer of Rachel mesh with the shade coefficient of 35%, which gives 40% of the area covered by fibers.^10^

Their water collection efficiency can be improved, for example, by amending fiber morphology,^11^ using steel harps,^12^ nature-inspired materials,^2^ and electrospun meshes which combine hydrophobic and hydrophilic polymers.^13^ The approaches in collecting water from fog can be found in electrostatically driven fog collectors that either use space charge injection^14^ or control the surface and bulk properties of polymeric materials.^15^ In nature, one excellent example of highly effective water collection is demonstrated by spiders, which use fiber webs with controlled surface charges^16^ and also special morphologies such as microcavities.^17^

The most common polymer surface modifications are metallization, ion implantation, cross-linking, and treatment by an external electric field, resulting in a multistep process that increases the overall production cost.^18^ Therefore, in our search for simple and cost-effective methods, we proposed electrospinning as one of the most promising techniques used to produce polymeric fibrous meshes. Importantly, this method makes it possible to tune surface and structural properties of the materials obtained during their production,^19^ compared to other manufacturing fiber methods without electric fields such as solution blow spinning^20^ or melt spinning.^21^ Electrospinning depends on many parameters such as relative humidity, temperature, applied voltage, and electrical polarity, which affect fiber structure and properties.^22^ Furthermore, the electrical polarity enables a control over surface chemistry and, consequently, surface potential. Electrospinning, with a positive or negative electrical polarity, causes an accumulation of electrical charges on the polymer jet surface, where molecules in polymer chains are repulsed or attracted, resulting in their reorientation.^23,24^ The surface potential of electrospun polymer fibers can be verified two ways, *via* direct measurement using Kelvin probe force microscopy (KPFM) and in liquids based on the zeta potential measurements.^25,26^

Among the wide range of polymers used for electrospinning processes, polycarbonate (PC) is an amorphous one with a rigid polymer chain structure, which is the result of both the aromatic character of the bisphenol A group and the partial double-bond character of the carbonate group.^27^ Because of this, it is possible to change the surface properties of electrospun fibers by reorienting the polymer chains *via* control of the electric field during electrospinning. This phenomenon makes it possible to change the surface properties of the fibers produced,^28^ which are explicitly verified with KPFM and zeta potential measurements. In this study, we investigated the surface properties of electrospun PC fibers and their effect on water collection efficiency. The surface chemistry and mechanical properties of PC fibers were controlled *via* electrical polarity and humidity levels during electrospinning to achieve one-step fabrication meshes for water harvesting applications. The experimental results were confirmed with a numerical simulation considering the surface potential effect on water droplets. The electrospun PC fibers exhibited an excellent mechanical stability and hydrophobic properties, which are desirable for the production of FWCs.

## Results and Discussion

### Morphology of PC Fibers

**Figure 1:**
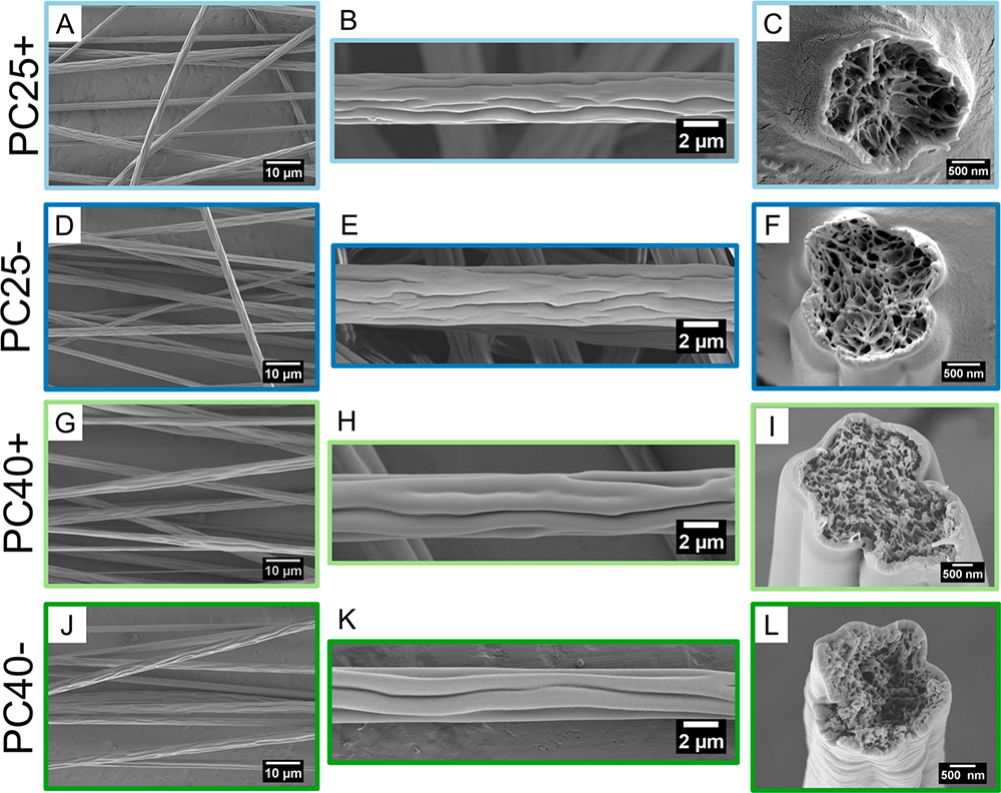
Scanning electron microscopy (SEM) micrographs of electrospun PC fibers produced with positive (A–C and G–I) and negative (D–F and J–L) electrical polarity at 25% (PC25+, PC25−) and 40% (PC40+, PC40−) RH with freeze-fracture indicating the voids present in them.

We developed PC electrospun meshes with positive (+) and negative (−) electrical polarity at 25% and 40% relative humidity (RH) for fog water harvesting; these meshes are identified in the text as PC25+, PC25–, PC40+, and PC40–. The surface morphology and cross-section investigation of the individual fibers revealed wrinkled surface structures and internal porosity, as shown in Figure 1. Average fiber diameters *D*_*f*_ for PC25+, PC25–, PC40+, and PC40– are 2.27 ± 0.48, 2.33 ± 0.51, 2.78 ± 0.54, and 2.77 ± 0.43 μm, respectively. The fiber diameter distribution results are illustrated in the histograms shown in Figure S1. The mean values of the pore fraction, pore size, and thickness of the PC meshes are in the range of 43.3–47.5%, 48–54 μm, and 142–153 μm for all samples, showing a very similar geometry. All results concerning morphology are shown in Table 1.

**Table 1:** Characteristic Average Values with Standard Deviation Related to Morphology, Surface, And Mechanical Properties of Fibers^a^

		PC25+	PC25–	PC40+	PC40–
morphology	fiber diameter (μm)	2.27 ± 0.48^a^	2.33 ± 0.51^a^	2.78 ± 0.54^b^	2.77 ± 0.43^b^
	fiber fraction in mesh (%)	51.46 ± 3.86^a^	55.81 ± 1.99^a^	54.67 ± 2.56^a^	56.71 ± 1.76^a^
	pore fraction (%)	47.54 ± 3.85^a^	44.19 ± 1.98^a^	45.32 ± 2.55^a^	43.28 ± 1.75^a^
	pore size (μm)	53.50 ± 3.20^a^	49.56 ± 2.99^a^	49.50 ± 2.18^a^	47.85 ± 4.21^a^
	mesh thickness (μm)	148 ± 21^a^	150 ± 27^a^	153 ± 21^a^	142 ± 27^a^
mechanical properties	max stress (MPa)	0.13 ± 0.02^a^	0.23 ± 0.01^b^	0.37 ± 0.06^c^	0.51 ± 0.01^d^
	strain at max stress (%)	24 ± 4^a^	60 ± 5^b^	240 ± 30^c^	203 ± 9^d^
	strain at failure (%)	281 ± 21^a^	282 ± 15^a^	374 ± 7^b^	351 ± 9^c^
	toughness (MJ·m^–3^)	16 ± 2^a^	36 ± 4^b^	119 ± 18^c^	180 ± 7^d^
surface properties	surface potential (mV)	71.5 ± 21.8^a^	461.0 ± 13.4^b^	210.6 ± 12.3^c^	365.7 ± 9.0^d^
	static contact angle (deg)	112 ± 4^a^	125 ± 4^b^	112 ± 3^a^	118 ± 4^b^
	roughness (μm)	15.02 ± 2.76^a^	16.09 ± 4.00^a^	14.24 ± 2.68^a^	15.05 ± 3.01^a^
	water collection rate (mg·cm^–2^·h^–1^)	56 ± 9^a^	70 ± 4^b^	60 ± 9^a^	72 ± 4^b^
	water collected after 180 min (mg·cm^–2^)	169 ± 1^a^	211 ± 4^b^	181 ± 1^c^	217 ± 7^b^

a Superscripts a–d indicate the statistical significance among each group.

PC fibers with positive and negative electric polarity had a similar average diameter; however, RH affected the result. Electrospinning at 25% RH led to lower average fiber diameter than at 40% RH. The morphology of the fibers was also different, as shown in Figure 1. In fibers produced with high humidity (PC40), grooves are directional in relation to the collector rotation axis and have different shapes and lengths than PC25 fibers. The differences in the morphology of PC fibers with 25% and 40% RH can be explained as being due to a variation in the evaporation dynamics of solvents in a polymer solution during electrospinning.^29^ RH has a strong influence on the surface and internal morphology, which can exhibit many different shapes and sizes.^30^ Electrospinning at high RH conditions causes the absorption and/or penetration of water into the polymer jet, thus leading to the formation of wrinkles on the surface, pores, and inner voids in the obtained fibers.^19^ Electrospinning at higher RH slows the evaporation of solvents from the polymer solution, resulting in a lower solidification rate of the polymer fibers.^31^ This mechanism was observed in the electrospinning of PVDF,^23^ where porous and nonporous morphologies were obtained at 60% and 30% RH. In our PC fibers, we noticed pores at both 25% and 40% RH. The role of the solvents used to prepare the polymer solution for electrospinning is also important, as it controls the water absorption rate.^32^ For the PC solution, we used two polar and water-soluble solvents, THF–DMF with high evaporation rate for THF.^33,34^ Additionally, we investigated a cross-section area of fibers, as shown in Figure 1 (C, F, I, L), indicating the fiber internal porosity. The voids in the fibers also occurred during a vapor-induced phase-separation mechanism,^35^ as water present in the atmosphere was adsorbed into the polymer jet and, after solidification, left imprints in the form of a porous core.

### DSC and FTIR

The thermal and spectroscopy analysis of PC samples were performed to verify any structural changes in electrospun fibers due to applied voltage polarity and relative humidity during electrospinning. Differential scanning calorimetry (DSC) heating scans are shown in Figure S2A. Crystallinity of samples and glass transition temperature were determined on the basis of heating curves; see eq S1 and Table S1. The crystallinity of the PC fibers was approximately 2.7% (PC25+), 2.3% (PC25−), 2.9% (PC40+), and 2.9% (PC40−). In contrast, PC films reach almost 20% of crystallinity; see Table S1. Additionally, the Fourier transform infrared spectroscopy (FT-IR) results are presented in Figure S2B, indicating peaks at 1161, 1188, and 1230 cm^–1^ for C–O bond stretching, at 1386 cm^–1^ due to C–H bending, at 1500 and 1684 cm^–1^ due to C=C stretching, and at 1770 cm^–1^ related to C=O.

DSC analysis showed no significant differences between the crystallinity of the obtained PC fibers, ranging from 2 to 3% of crystallinity; see Figure S2 and Table S1. Such low crystallinity of the fibers is due to the amorphous structure of PC. Similar results of very low crystallinity were also obtained for other electrospun amorphous polymer fibers.^36^ FT-IR results confirmed no differences between the fibers produced with RH 25 or 40% with ± electrical polarities. The variation in absorbance for individual wavelengths from the samples tested was typically around 1%, which is not a significant difference that requires further consideration. The peaks for the individual chemical bonds contained in the PC are in the correct positions indicating that the same material was used for each of the fiber samples. However, PC films spectra was slightly shifted depending of wavelength, which also confirmed an influence of electrical field on PC polymer structure. DSC and FT-IR show no structural changes in the PC samples of electrospun fibers.

### Mechanical Testing

**Figure 2:**
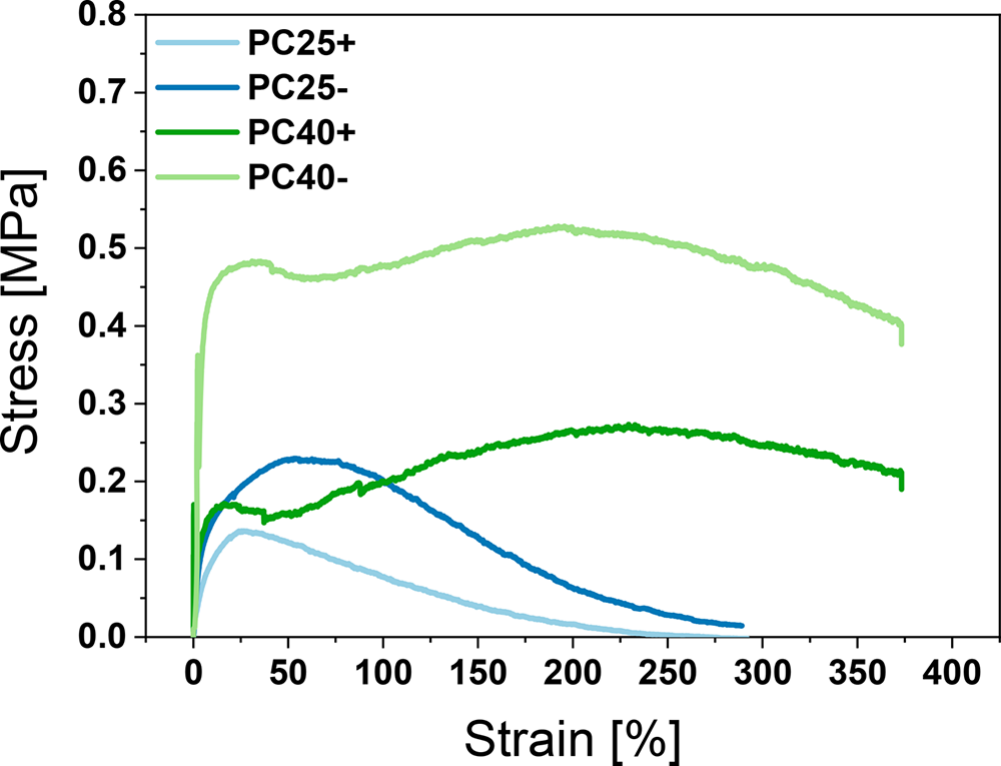
Representative stress–strain curves from the tensile testing of PC meshes.

The mechanical properties of electrospun PC meshes were tested using a tensile module, and representative results are shown in the form of stress–strain curves; see Figure 2, data in Figure S3, and summary in Table 1. For PC25– fibers, we observed ∼1.76 times higher tensile stress, ∼2.25 times greater toughness, and ∼2.5 times greater elongation at maximum stress than with PC25+ meshes. For fibers produced with higher RH, PC40 showed a relation between electrical polarities similar to that of PC25. Fibers produced with positive electrical polarity at higher humidity level (PC40+) showed a lower tensile stress (0.37 ± 0.06 MPa) and toughness (119 ± 18 MJ·m^–3^) and a higher strain at maxmum stress (240 ± 30%) than PC40– (0.51 ± 0.01 MPa, 180 ± 7 MJ·m^–3^, 203 ± 9%). Moreover, the effect of higher humidity on the mechanical properties of fibers can be observed.

The tensile test results indicate a significant improvement in the mechanical properties of PC fibers when using negative voltage polarity at high RH during electrospinning, as confirmed previously with electrospun PMMA fibers.^36^ The increased mechanical properties of the PC samples produced with positive and negative electrical polarity are related to changes in the electric force strength needed to elongate the polymer jet during electrospinning. The effect of humidity on the mechanical properties of electrospun meshes was investigated so far due to differences in the internal structure and the number and sizes of pores.^37,38^ That effect can be seen in our samples in Figure 1 and is caused by the water molecules in the atmosphere during electrospinning. For random arrangement of fibers in meshes, their mechanical performance depends on the mechanical properties of individual fibers.^39^ The random orientation of fibers in meshes causes stress delocalization and enhancement of mechanical performances due to the interaction among the fibers,^40^ indicating the importance of individual fiber surface properties.

### Surface Chemistry

**Figure 3:**
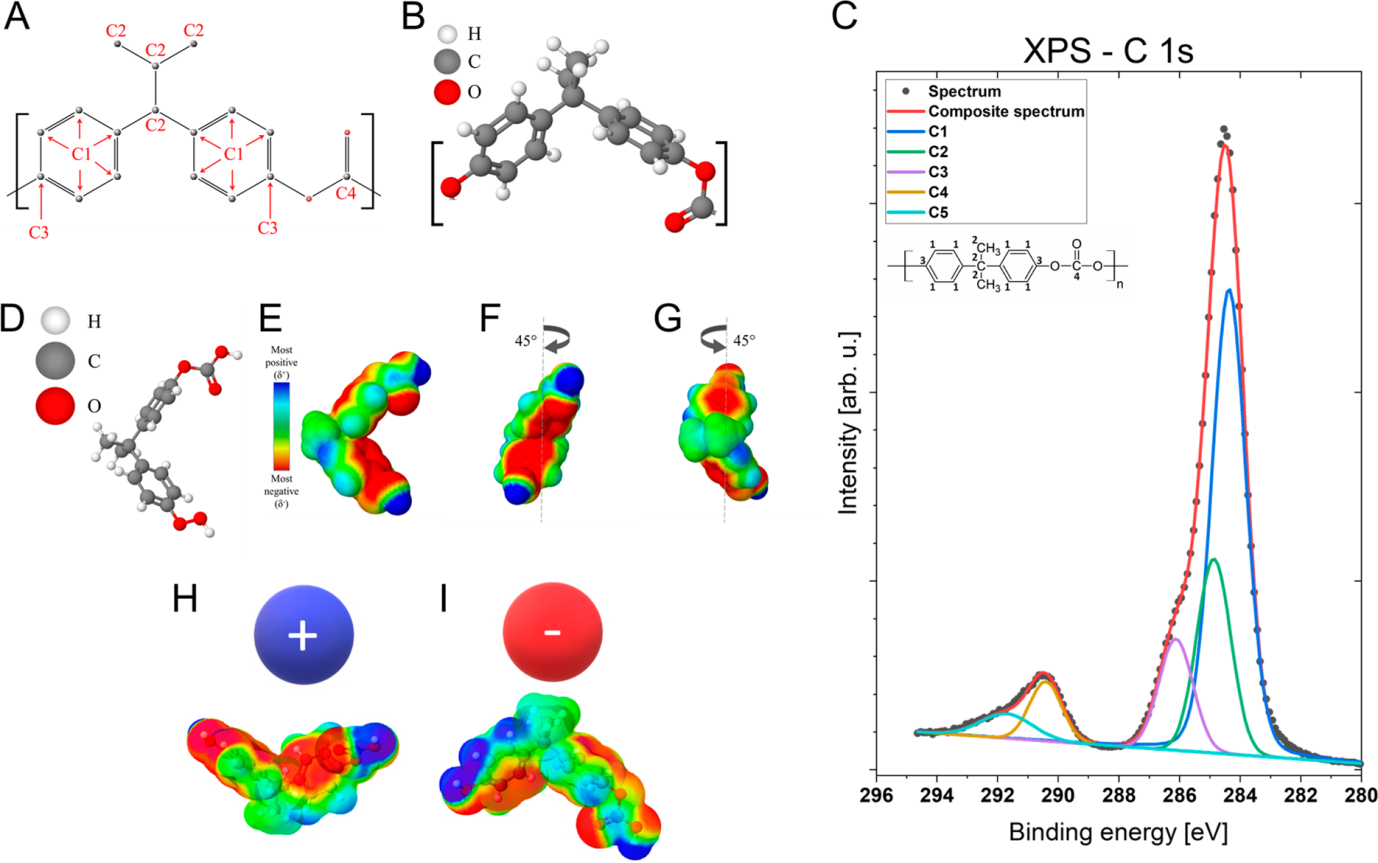
(A, B) are 2D/3D schematic images of the single unit of the PC polymer chain structure; symbols: C1–4 refer to the measured atomic % of each carbon in PC by XPS. (C) Representative XPS spectrum for C 1s region for PC film. (D) Model of the PC polymer structural unit used to generate an electrostatic potential map. (E) Electrostatic potential map of a single PC unit. (F, G) 45° rotation in the *Y*-axis to indicate the electrostatic surface potential in different positions of the molecule. (H, I) Schematic reorientation model of the single-unit PC polymer chain, with the applied positive and negative electrical polarity during electrospinning.

The surface chemistry of the electrospun PC fibers and films was analyzed using angle-resolved X-ray photoelectron spectroscopy (ARXPS). The angle between the sample and the analyzer was set to 10° in order to obtain information from a 1–2 nm depth in the fiber surface. The atomic concentrations of each chemical state are shown in Table 2, while 2D/3D schematic images of the single unit of the PC polymer chain structure and representative XPS spectrum for C 1s region for PC film are presented in Figure 3. The positions of fitted components are based on previous studies.^41^ ARXPS results showed significant differences in the chemical composition on the PC fiber surface between samples produced with positive and negative electrical polarity, but also at 25% and 40% RH. The lower contents of C1 (45.5 and 40.5 atom % for PC25 and PC40, respectively) in the benzene aromatic ring and C2 in the alkyl group (17.3 atom % (PC25) and 15.4 atom % (PC40)) were observed for positive electrical polarity. Interestingly, with a positive electrical polarity we observed higher C3 (originating from oxygen to benzene ring bonds) contents, namely 29.2 and 38.2 atom % for PC25+ and PC40+, respectively. The spin-coated PC film showed different C1, C2, and C3 surface contents compared to the electrospun PC fibers, as it is produced without electric field effects.

**Table 2:**
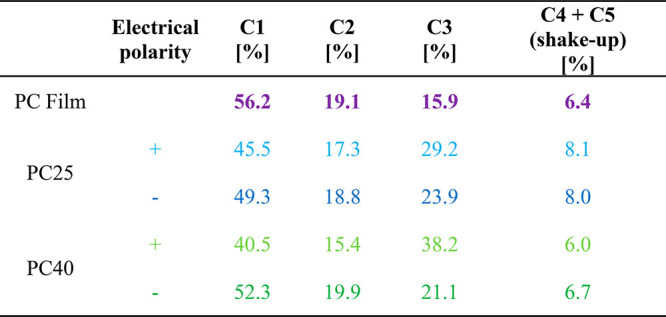
ARXPS Results from the Low Take-Off Measurements (10°) Shown as Atomic % for PC Film and Electrospun Fibers Produced with Positive (PC+) and Negative (PC−) Electrical Polarity

The differences in the surface chemistry of the PC film samples treated by external electric fields such as corona, glow discharge, *etc*. were observed in several studies.^42−44^ These indicate that the electric field and charges affect the chemical composition of the surface of electrospun PC fibers. As we mentioned earlier, the spin-coated PC film showed C1, C2, and C3 surface contents that were completely different from those of the electrospun PC fibers, thus demonstrating that a molecular reorientation occurred during electrospinning. The reorientation of polymer chains during electrospinning with positive and negative electrical polarities, due to the charges accumulated on the polymer jet surface, caused carbon and oxygen elements in the polymer chains to be attracted or repelled.^24,28^ Importantly, in PC fibers we observed clear changes in surface chemistry with a difference in C3 content as high as 20%. Figure 3E shows the 3D model of the structure and the electrostatic potential map of a single PC polymer chain unit. The mapping indicates negative potential regions close to benzene rings (Figure 3E–G, red color). Electrons in the covalent bonds of the benzene rings form a quadrupole moment due to the stronger electronegativity of sp2 carbons compared to hydrogen atoms. This quadrupole generates a negative potential on both faces of the π system and a negative charge inside the aromatic ring.^45^ In addition, oxygen is attached to the aromatic rings and carbonate group, thus generating an additional negative potential. Moreover, the PC structural unit is characterized by a rigid polymer chain caused by the presence of a carbonate group (−CO_3_) and benzene rings,^46^ thus limiting unnecessary movements of the unit. Figure 3H–I shows the schematic model of the possible reorientation of a PC single polymer unit. The presence of negatively charged molecules in a PC polymer chain causes reorientation, when a negative electrical polarity is applied. The negatively charged benzene ring and oxygen regions are repelled, causing the chain to rotate, and decreasing the C3 contents on the polymer jet surface during electrospinning. Conversely, with a positive electrical polarity, those negative regions are attracted to the surface, increasing the C3 content on the surface of the PC fibers. We also observed the RH effect on chemical composition regardless of electrical polarity; see the contents of C1, C2, and C3 in Table 2. The changes in the surface chemistry of PC samples produced with 25% and 40% RH are caused by the overlapping of two effects: charge density and solvent evaporation rate during electrospinning. Importantly, humidity affects the distribution of electrical charges on the surface of a polymer jet during electrospinning. A low RH leads to a high charge density as there are no water molecules causing a discharge of the polymer jet.^47^ As we mentioned in the discussion on fiber morphology, the low humidity increases the evaporation rate. To summarize, with low relative humidity the charge density is higher, as the evaporation rate of solvent is faster; therefore, the dynamics of chain reorientation and the time it takes for the fibers to solidify are different. High relative humidity leads to slow solidification, giving the polymer chains the time necessary to complete reorientation, thus causing the surface chemistry composition to change. Our results clearly indicate the position of carbon (C1 and C3) in the aromatic ring and also of C2 in relation to the fiber surface. Importantly, it can be controlled not only by the applied electric polarity, but also *via* the humidity level.

In opposition to surface chemistry verified with XPS analysis, DSC and FT-IR show no structural changes in the PC samples. Surface chemistry of PC fibers varies due to electrospinning with different electrical polarities, as the charges accumulating on the fiber surface interact with molecules at about ∼2 nm depth of the material surface.^24,48^ The DSC and FT-IR results are from bulk of the investigated material, as the entire volume of the sample is measured and any surface chemistry changes cause only marginal differences in the crystallinity or absorbance results.

### Surface and Zeta Potential

**Figure 4:**
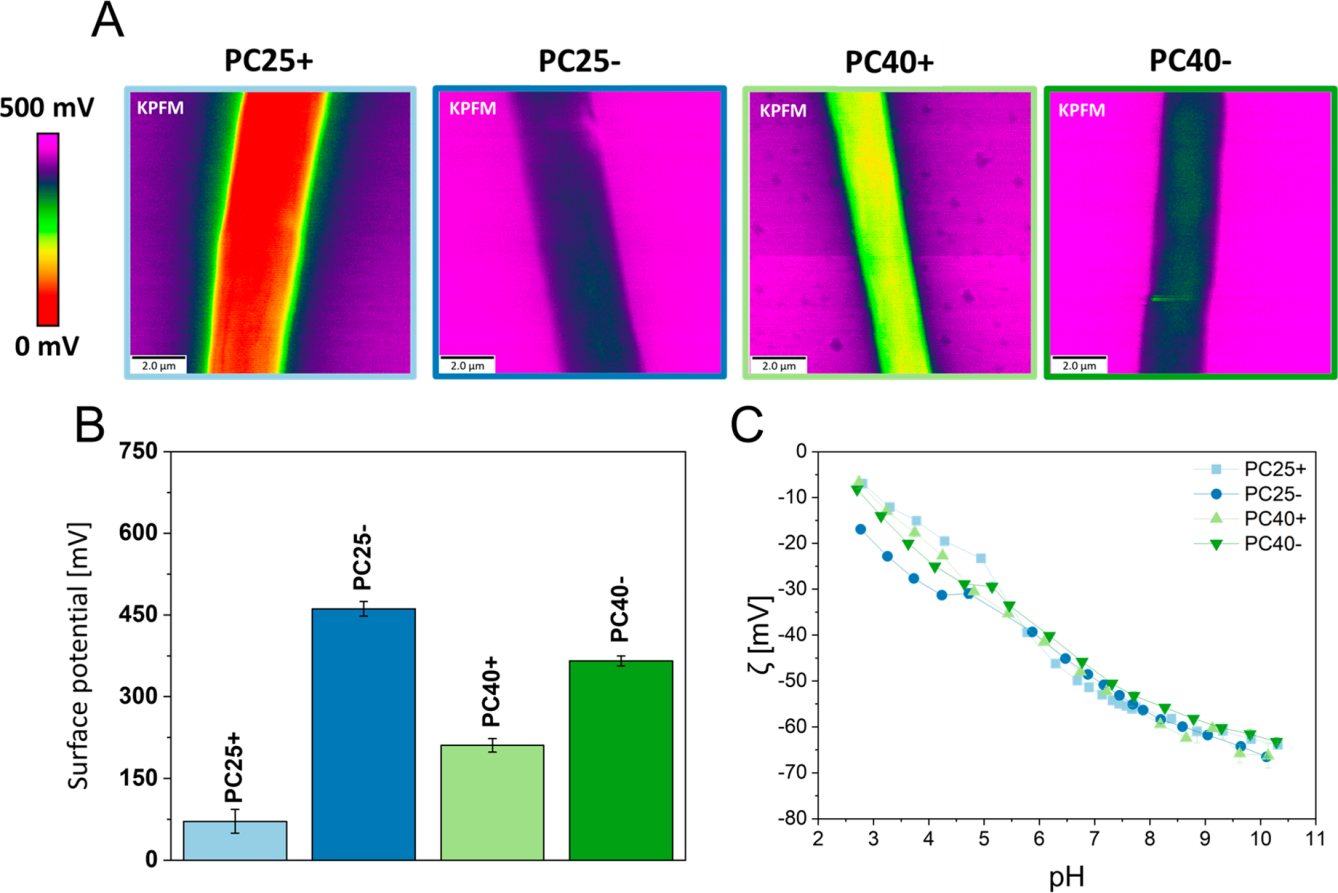
Surface potential and zeta potential characterization of electrospun PC fibers produced with positive (PC+) and negative (PC−) electrical polarity: (A) KPFM scans, (B) surface potential results for PC samples measured by KPFM, (C) titration curves of PC mesh in the function for pH in KCl solution indicating zeta potential values of PC meshes.

The surface potential of the PC fibers and film were examined using KPFM as shown in Figure 4A,B. The value of 71.5 ± 21.8 mV for PC25+ was almost ∼6.5 times lower than for PC25–, showing 461.0 ± 13.4 mV. The difference between PC40+ and PC40– was only ∼1.7 times lower; see Figure 4B. The value of the surface potential of PC40+ was three times higher than PC25+, but for PC40– a reverse effect was observed and the surface potential was 1.25 times lower than PC25–. PC film showed completely different results in comparison to electrospun fibers and was −573.2 ± 3.7 mV; see data in the Figure S4. The topography from atomic force microscopy (AFM) is shown in Figure S4.

The zeta potential of PC fibers was measured using a standard KCl solution to investigate zeta potential evolution *vs* pH; see Figure 4 C. The titration curve was presented in the range from 2.5 to 10 pH. PC25– samples showed the highest zeta potential (∼−17 to −39 mV) in the range from 2.5 pH to 5.5 pH over PC40– (∼−8 to −33 mV), PC40+ (∼−7 to −35 mV), PC25+ (∼−7 to −39 mV). Above 7 pH, the zeta potential value was similar for all fibrous samples and is approximately −45 to −64 mV (from 6 to 10 pH). The polymer film also showed differences in zeta potentials (as well as in the case of the electric surface potential), ranging from +2 to −25 mV (2.5 to 5.5 pH) and from −20 to −22 mV (6 to 10 pH); see Figure S5.

The surface chemistry affects the surface potential and zeta potential, as was also previously seen when electrospinning PCL, PMMA, and PVDF.^23,28,48^ The surface potential value increased with negative electrical polarity. Ahn *et al.* obtained similar results in the surface potential of PC filters for water treatment but attributed them to changes in the voltage used in electrospinning.^49^ The surface potential in PC fibers is evidently related to the surface chemistry, which is controlled by the electrical polarity. Additionally, the surface potential value changed with the RH regardless of the electrical polarity. Zeta potentials for PC membranes showed the greatest differences at pH values from 2.5 to 6, which is in the range of water harvested from typical fogs around the world (3.5–6 pH).^50,51^ The highest level of zeta potential was observed for PC25+, with the lowest surface charge measured with KPFM. Interestingly, for pH values above 7, the difference in the zeta potential of the tested samples ceased to show significant changes. This is due to the fact that the poor resistance of PC to alkaline environments leads to the release of bisphenol A from its polymer chains, and thus, the chemical surface conformation of the fibers is changed.^52^

### Numerical Simulation of Surface Potential Effect on Water Droplet

**Figure 5:**
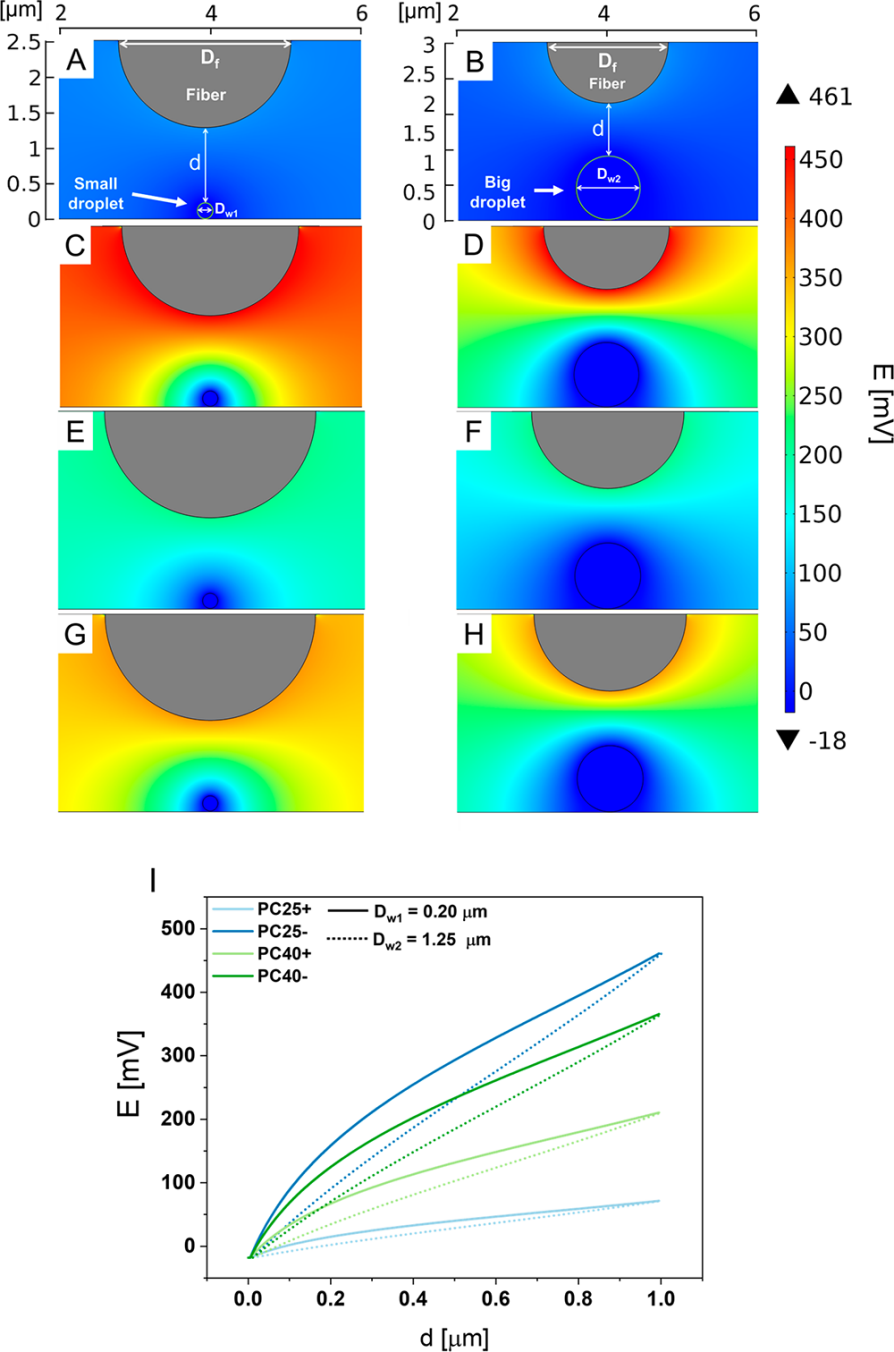
Electric potential distribution map between PC25+ (A, B), PC25– (C, D), PC40+ (E, F), PC40– (G, H), and water droplet with diameters of *D*_*w1*_ = 0.20 μm (A, C, E, G) and *D*_*w2*_ = 1.25 μm (B, D, F, H). Distance between water droplet and fiber is *d* = 1.00 μm. (I) Relation between electrical potential *E* (taken from line along *d*) to distance d from surface of droplet to fiber for small (*D*_*w*_ = 0.20 μm, straight line) and big water drop (*D*_*w*_ = 1.25 μm, dotted line).

The purpose of this study is to verify the effect of surface potential of polymer fibers on water collection from fog which is imitated by the humidifier producing the droplets sizes in the range from 0.20 to 1.25 μm.^53,54^ The surface potential of water droplet is approximately −18 mV,^55^ and the surface potential of the fiber we measured with KPFM from 72 to 461 mV, as shown in Figure 4. We assume that water droplets should be attracted to the surface of PC fibers that have the greatest potential. To verify the effect of electrostatic potential difference between the water droplet and the fiber surface a simple numerical model was prepared. The simulations were performed for two boundary droplet diameters referring to the mentioned droplet sizes, which were *D*_*w1*_ = 0.20 μm and *D*_*w2*_ = 1.25 μm,^53,54^ and for the measured PC fiber diameter *D*_*f*_; see Table 1 and Figure S1. In the numerical model the distance between the droplet and the fiber surface was *d* = 1.00 μm, as shown in Figure 5. In addition, we extracted electric field data in a straight line from the surface of the droplet (see Figure 5A, *d*) to the fiber surface to correlate the relation between electrical potential *E* to distance *d*; see Figure 5I. In Figure 5A,B, we observe that for fibers PC25+ with the lowest surface potential value the gradient is the weakest. On the other hand, fibers PC25– and PC40– characterized by the highest surface potentials show the strongest electric potential gradient (Figure 5C,D,G–H). Figure 5I indicates that the size of water droplet is important in the electrostatic interactions between PC fibers. For the larger droplet (*D*_*w2*_ = 1.25 μm), the curve takes the shape of a near a straight line, where for a smaller drop it is nonlinear.

### Water Harvesting and Wetting Properties

**Figure 6:**
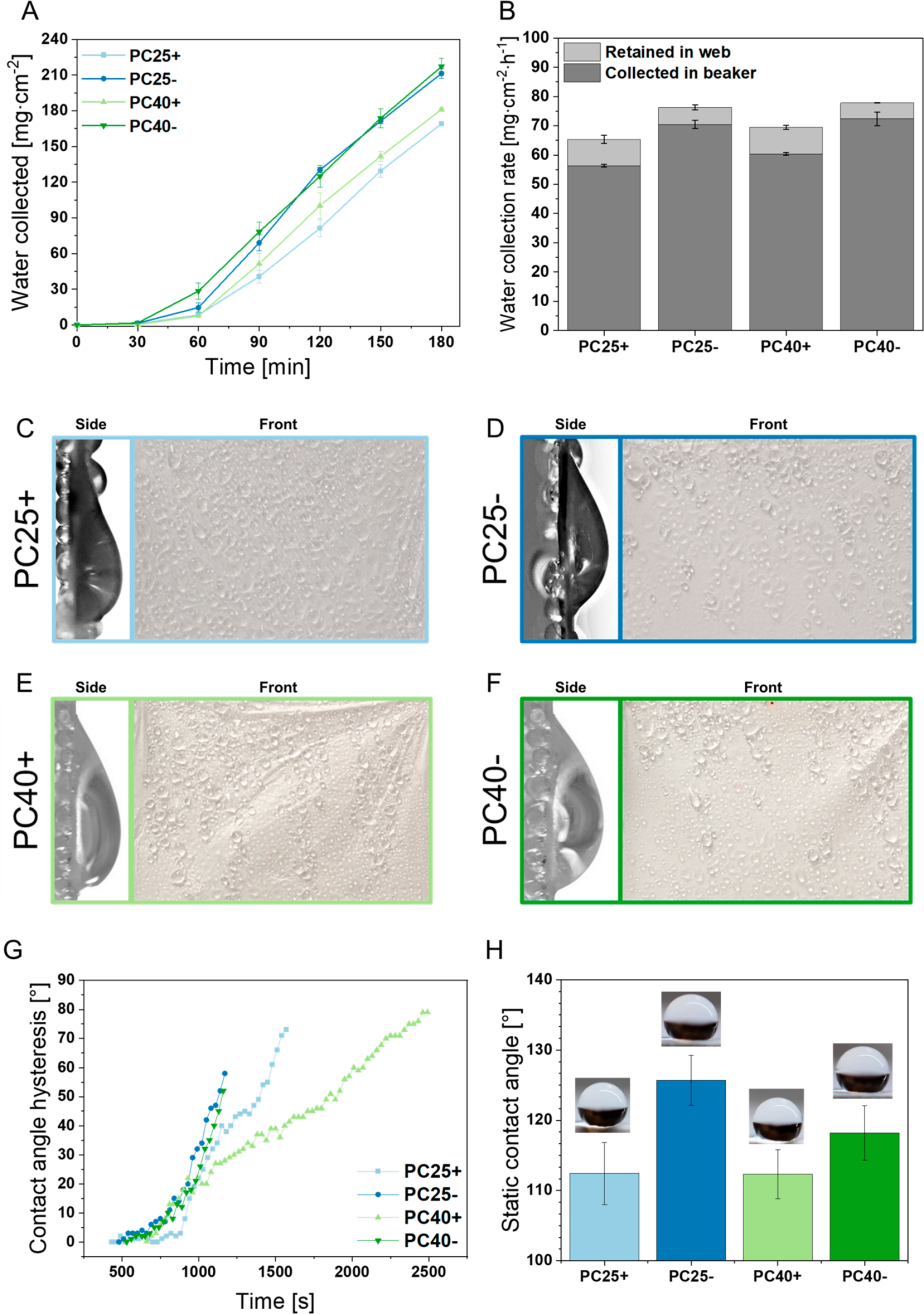
Water collection results for electrospun PC fibers produced with positive (PC+) and negative (PC−) electrical polarity: (A) fog water collection in 3 h test and (B) water collected rate per hour. (C–F) images of deposited water droplets on vertical electrospun PC meshes before flowing down into the beaker in side view and in the front view collected droplets after 90 min of fog water on the mesh surface. (G) Graph indicating the changes in contact angle hysteresis on meshes in time. (H) is a static contact angle measurement with representative images of deposited water droplets on the meshes placed horizontally.

The meshes with positive electrical polarity collected ∼18% less water into the beaker per hour than negative electrical polarity meshes in the same experimental conditions; see Figure 6A,B. This difference was also observed in the number of droplets deposited from fog after 90 min of test (Figure 6C–F). Additionally, the hysteresis of the dynamic water contact angle was measured on vertical PC meshes, as shown in Figure 6G. The PC meshes with negative electrical polarity (PC25–, PC40−) had a lower contact angle hysteresis (∼55°) right before the water drops fell into the beaker (after ∼1200 s) and earlier. PC25+ and PC40+ meshes, reaching almost 70°, show a fall after ∼1500 s, and at 80° show a fall after ∼2500 s. These differences in the contact angle hysteresis are directly correlated with the water collection efficiency, as shown in Figures 6G,H and Figure S6.

PC meshes produced with negative electrical polarity are characterized by a higher water collection capability, compared to those produced with positive electrical polarity; see Figure 6A,B. The differences were also noted in static water contact angles, as shown in Figure 6H. In collecting water from fog, the permeability of the membrane—which is related to the pore fraction, pore size, and fiber diameter—is crucial and can drastically change the mesh efficiency.^56^ In FWC it is correlated with the shade coefficient.^8^ As shown in Figure 1 and Table 1, both types of samples have a grooved morphology, without any particular difference in fiber diameter, pore size, or fraction. The grooved morphology enhanced the hydrophobicity of polymer fibers.^11,57^

Surface chemistry and surface potential significantly affect the interaction between the solid surface layer of the material and water.^18^ It is possible that the conformation of the surface molecules on which the water is deposited arranges its dipoles. Consequently, water molecules can be repelled from surfaces on an atomic scale.^58,59^ Dreier *et al.* presented simulations where the surface potential also affects the conformation of water dipoles and scales by surface charges, which can be measured with zeta potential.^60^ Water dipoles are able to reorient, thus essentially changing the shape of the droplets following the increase in water collection for PC- samples (Figure 6B) by reducing the contact angle hysteresis (Figure 6G). Clearly, the electric surface potential of PC fibers controls the deposition of fog droplets. As previously discussed, the electrospinning with positive and negative voltage polarity changes the surface free energy of polymer fibers following with the differences in wetting of individual fibers.^24^

**Figure 7:**
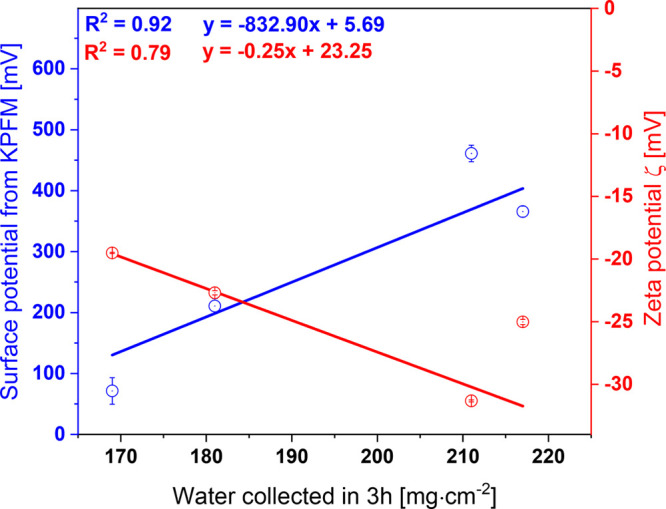
Correlation between the surface and zeta potential (pH = 4.5) with fog water collection in 3 h.

In Figure 7, a correlation between the surface potential and zeta potential (pH = 4.5) to water collection is presented showing the increase of collected water from PC fibers characterized with the highest surface potential directly measure using KPFM. This trend is kept for the lowest zeta potentials as their values are opposite to the surface potential measured with the KPFM; thus, the water collection increases with decrease of the zeta potential. The numerical model of the electrostatic potential interactions between water and solid, see Figure 5, indicates that fibers with highest surface potential (PC25– and PC40−) exhibit the greatest ability to collect water due to the highest potential difference and thus the strongest electrostatic force between droplet of water and surface of PC fiber. Samples PC25+ with the lowest surface potential and the weakest electric potential gradient collected the smallest amount of the fog water, see Figure 5A,B and Figure 6B. Additionally, the results shown in Figure 5I suggest that the size of droplet plays a significant role in electrostatic attraction of water and following with the water collection rate. The highest KPFM potential of PC25– and PC40– fibers is able to attract also the smaller fraction of droplets that can be captured by the PC mesh.

In relation to the theoretical studies of the surface properties of materials, many of them are focused on wettability;^61−66^ however, only a few models include the effect of surface potential on the wetting properties.^67−72^ Wang *et al.* showed that surface charge difference causes a change of friction coefficient between two-dimensional material and water surfaces.^68^ The theoretical investigation of electrical charges influence on dielectric surfaces on wetting properties was also investigated by Liu *et al.*(72) exploring the electrowetting mechanisms,^66^ where voltage is applied to surface with water droplet.^69^ The surface charge can be transferred to droplets allowing their movement or sliding and increasing drop number.^73^ Importantly, the electrification of water droplet by sliding on polymer surfaces depends on the velocity and the conductivity of droplets causing the charge exchange between polymer surface and water.^74^ The electrification between hydrophobic dielectric polymer surfaces and water has a potential to produce the electrostatic energy being simply a hydroelectric generator.^75^

## Conclusions

We propose a simple one-step application for increasing the applicability and effectiveness of electrospun meshes as fog collectors. Water collection efficiency of electrospun meshes depends on the surface potential and surface chemistry of the fibers, which can be improved by adjusting electrical polarity and relative humidity during electrospinning. PC meshes produced with negative electrical polarity at humidity reaching 40%, showed a greater water collection rate than other electrospun fog collectors (∼46–145% higher efficiency)^13,56,76^ under the same experimental conditions. Additionally, PC fibers with higher surface potential are able to attract electrostatically smaller droplets, what has been confirmed with the numerical simulations. A deep understanding of water interaction with the designed surface properties of fibers is crucial for the development of materials capable of tackling global challenges, such as the access to clean water.

## Experimental Methods

### Electrospinning and Spin-Coating of Polycarbonate

To obtain a 24 wt % solution, polycarbonate (Makrolon 3108, Goodfellow GmbH, Germany) was dissolved in a mixture of *N*,*N*-dimethylformamide (DMF, Sigma-Aldrich, UK) and tetrahydrofuran (THF, Sigma-Aldrich, UK) in a 1:1 weight ratio. The solution was stirred at 700 rpm for 2.5 h on a hot plate set at 60 °C (IKA RCT basic, Germany). PC fibers were produced *via* electrospinning (apparatus EC-DIG with climate control, IME Technologies, The Netherlands) at *T* = 25 °C and 25% (PC25) and 40% (PC40) relative humidity. A voltage of 16 kV with positive (PC25+ and PC40+) and negative polarity (PC25– and PC40−) was applied to the needle kept at a distance of 24 cm from the grounded rotating drum at 10 rpm that was used as a collector to produce random fibers. The flow rate was set to 0.03 mL·min^–1^. Electrospinning time was 30 min for all samples. We selected two levels of relative humidity - the minimum (25%) and maximum (40%) at which electrospinning processes were stable. The samples were deposited on baking paper for SEM analysis and on Au-coated silicon wafer for XPS and KPFM analyses. PC films were spin-coated on a 11 × 11 mm glass and silicon wafers (L2001A v.3, Ossila, UK) for 60 s at a rotation speed of 2000 rpm, at RH = 40% and *T* = 25 °C, after placing 0.1 mL of the same PC solution that was used for electrospinning.

### Surface Morphology

PC sample morphologies and cross sections were investigated using SEM (Merlin Gemini II, ZEISS, Germany) at 3 kV accelerating voltage, 110 pA current, and a working distance between 4 and 9 mm. Prior to the SEM analysis, samples were coated with a 5 nm thick Au layer using a sputter coater (Quorum Q150RS, Quorum Technologies Ltd., UK). Fiber diameters were measured from SEM micrographs using ImageJ software (version 1.51, Fiji, USA). The average *D*_f_ values were calculated from 100 measurements, and the error was based on the standard deviation. The data from the fiber diameter measurements were expressed as the arithmetic average ± standard deviation (SD). The cross-section imaging of PC fibers was achieved by the freeze fracture method, where samples were soaked in liquid N_2_ for 5 min and cracked using a scalpel prior to SEM imaging.^13,23^ The pore fraction and size were calculated with the *analyze particles* function in ImageJ software (version 1.51, Fiji, USA).

### Roughness of Electrospun Meshes

A profilometry study was conducted using an optical profiler (Veeco, WykoNT9300, USA) at the following settings: objective (20 × ), field-of-view multiplier (0.55×), and sampling area (910 nm), in order to obtain the roughness average (*R*_a_). *R*_a_ is the arithmetic mean of the absolute values of the surface departures from the mean plane, which is used to describe the roughness of the measured area.^77^

### Thermal Analysis of Electrospun Samples

The samples were measured at a scanning rate of 10 °C·min^–1^ using differential scanning calorimeter (DSC, TA Instruments, Q2000, USA) operating under a nitrogen purge. The cut pieces of from PC meshes and film (*ca*. 2 mg) were sealed into T-zero pans (TA Instruments, USA) prior to the measurements heated from 25 to 280 °C, at a rate of 10 K·min^–1^. DSC data and heating scans are shown in Table S1 and Figure S2A.

IR spectra were obtained using a IR spectrometer (Bruker, Tensor 27, USA) equipped with an attenuated total internal reflection attachment. All spectra have been normalized, atmospheric corrected, offset and background subtracted for visual clarity, and are shown in Figure S2B.

### Mechanical Testing of PC Fiber Meshes

During electrospinning, PC fibers were deposited directly onto the surface of laser-cut 20 × 8 mm rectangular paper frames, which were later used in a tensile module (1 N cell, Kammrath & Weiss, Germany) at *T* = 24 °C and RH = 40%, using the extension rate 20 μm·s^–1^. Average values were calculated from 5 tests for the maximum stress, toughness, and strain at maximum stress. The stress was analyzed as a force measured by the tensile module to the initial cross-sectional area of the electrospun fiber mat. To measure the thickness of the membrane and film their cross-section was obtained *via* freeze facture described in Surface Morphology section and imaged with SEM, as showed in the Figure S7. The measurements were taken using ImageJ (version 1.51, Fiji, USA).

### Surface Chemistry

The surface chemistry of PC samples was investigated using an X-ray spectroscopy system (XPS, VersaProbe II, PHI, USA) with monochromatic radiation from aluminum Kα (1486.6 eV) focused to a 100 μm spot and at a 10° photoelectron takeoff angle. The pass energy in the analyzer was set to 23.50 eV to obtain high-energy resolution spectra for the C 1s region. The fibers were analyzed perpendicularly to the analyzer inlet to prevent the influence of the cylindrical surface of the material on the results obtained. In order to maintain a constant sample surface potential, regardless of the sample conductivity, a dual-beam charge compensation with 7 eV Ar+ ions and 1 eV electrons was used. The operating pressure in the analytical chamber was 4 × 10^–9^ mbar. The spectra obtained were deconvoluted using the MultiPak software (PHI, Chigasaki, Japan). The Shirley method was used to subtract the XPS spectrum background. In Table 2, the percentages are calculated as a percent of each line area to sum of all line’s areas. The C4 + C5 is reported together due to the fact of overlapping shakeup component (which for clarity are fitted on presented spectra as one broad peak but should be fitted with four to five smaller lines) and O–(C=O)–O component. The C5 component is the so-called “shake-up” satellite which appears on the high binding energy side of the main photoelectron line.

### 3D Models of Polycarbonate Chemical Structures

Chemical structures and the molecular electrostatic potential map of a PC chain single unit were visualized using an open-source molecular builder and visualization tool – Avogadro (version 1.2.0m, USA).^78^

### Characterization of Surface Potential and Zeta Potential

AFM was used for KPFM and topography measurements by means of Bruker multimode 8 (Bruker, USA) using MESP-RC-V2 tips (Bruker, USA) with a spring constant of 5 N·m^–1^. Calculations of the average surface potential concerned 3 different regions on the KPFM scan according to the previously reported protocols.^28^ AFM topography results are shown in Figure S4.

The zeta potential of the PC fibers was measured using an electrokinetic analyzer for solid surfaces (SurPASS 3 Eco, Anton Paar, Austria) with an adjustable gap cell. Titration curves were obtained by zeta potential measurements in a 0.01 M KCl electrolyte solution. The pH variation from 2.7 to 10 was obtained with a progressive addition of 0.05 M HCl or 0.05 M NaOH to the solution for the acidic and basic regions, respectively. Titration curves are presented as average value with error bars calculated from 5 tests, as shown in Figures 1K and Figure S5.

### Numerical Simulation of Surface Potential Effect on Water Droplet

Distribution of electric potential between single fibers and a water droplet was simulated using COMSOL Mutliphysics (version 5.6, COMSOL Inc., Sweden). In the mathematical 2D model, half of cross-section of the fiber and the whole water droplet were considered; see Figure 5. According to the literature, the water droplet diameter *D*_*w*_ is in the range 0.20–1.25 μm;^53^ therefore, in our simulation we used these two extreme values. In our simulation we used a value of −18 mV^55^ for water surface potential, which depends on experimental setup, measurement methods, and computer model.^55^ The distance between the fiber and the water droplet was set to *d* = 1 μm, as the focus is on the single fiber and the single water droplet interaction. The computing domain was 8 μm width, and its height depended on the fiber radius and water droplet size; thus, the height of the model was in the range of 2.34–3.59 μm. In Figure 5 we show the most important part of simulation is between 2 and 6 μm of the total domain of 8 μm. The calculations were resolved as stationary study. The external domain boundaries were taken as zero charge. The water relative permittivity was taken as 80.2 and relative permittivity of air (filling space between the fiber and the water droplet) as 1.0. The example of computational mesh is shown in Figure S8. The triangle mesh parameters were closed in ranges: elements number 186454–275773, element size 0.0032–0.0160 μm, maximum growth rate 1.1, curvature factor 0.25, minimum orthogonal quality 0.5355 and average quality 0.9389–0.9428. The numerical simulation was calculated with a dielectric model of polarization from the following equations
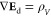
1
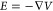
2where *E*_d_ is the electric displacement field (C·m^–2^), *ρ*_V_ is charge density (C·m^–2^), *E* is the electric field (V·m^–1^), and *V* is the electric potential (V). For the plots drawn we used data from the *d* line driven vertically across the calculated model as shown in Figure 5A.

### Wetting Properties

Advancing contact angles on randomly oriented PC electrospun fibers, deposited on glass slides, were measured using deionized (DI) water (pH ∼ 5, surface tension γ = 72.2 mJ·m^–2^, Spring 5UV purification system – Hydrolab, Poland). The images of droplets were taken using a DSLR camera (EOS 700D, lens EF-S 60 mm f/2.8 Macro USM, Canon, Japan) after 5 s from the deposition of 3 μL droplets on the samples. Experiments were carried out at *T* = 25 °C and RH = 45%. Contact angles were measured for 10 different droplets deposited on fibers by using a drop shape analysis plug-in in ImageJ (version 1.51, Fiji, USA).

### Fog Collection Experiments

PC meshes were cut to 10 × 10 cm squares. Next, samples were placed on a special stand in a vapor stream, 6 cm away from and perpendicular to the fog outlet, where there was a humidity range of 95% to 99%. The water collection rates were measured in laboratory conditions at *T* = 24 °C using a conventional water humidifier (Beurer GmbH, Germany) with deionized (DI) water (pH = 4.8, surface tension γ = 72.2 mJ·m^–2^, Spring 5UV purification system – Hydrolab, Poland) as indicated in a previous study.^13^ Fog flow and velocity were 400 mL·h^–1^ and 0.19 m·s^–1^, respectively. The water collected on meshes was drained into the glass beaker placed underneath, which was weighed every 30 min over a 3 h period. The scheme of experimental setup was illustrated in previous study.^13^ The collected water was calculated as previously described,^13,56,76^ by the water mass obtained per mesh area. The 1 h water collection rate was calculated as the water collected during deposition, divided by the total number of experimental hours.

Dynamic water contact angles were measured in a vertical position on the droplets growing on random PC fibers during the fog water collection experiment. The mesh width was limited to 1 cm, and images were taken every 5 s from the start of droplet growth until they fell into the beaker. Contact angle hysteresis was calculated by subtraction of the advancing from the receding contact angle.

### Statistical Analyses

Fiber morphology values, surface potential, zeta potential, and mechanical properties were statistically analyzed with OrginPro (2020 SR1, OriginLab, USA) software, using Student’s *t* test. For all tests, the significance was set at *p* < 0.05. Data are expressed as the arithmetic average ± standard deviation (SD). All average values are summarized in Table 1.

## Supporting Information Available

Histograms of PC fiber diameter distribution (Figure S1); DSC heating scans and FT-IR spectra (Figure S2); equation used for determination of crystallinity of PC samples (eq S1); crystallinity and heat of melting for PC film and electrospun fibers (Table S1); stress–strain curves of PC samples (Figure S3); AFM topography of PC fibers and KPFM results for PC film (Figure S4); titration curve of the PC film (Figure S5); image of water droplets deposited on a PC film (Figure S6); images of cross-section thickness of PC fibers and film (Figure S7); example mesh of model used for numerical simulation (Figure S8) (PDF)
