# Supplementary material for: Surface Potential Driven Water Harvesting from Fog
Source: ACS Nano. 2021 Apr 26;15(5):8848–59. doi: 10.1021/acsnano.1c01437 (PMC8158858; doi:10.1021/acsnano.1c01437)
Supplement: Supplementary file 1 — nn1c01437_si_001.pdf [file nn1c01437_si_001.pdf]

Supporting Information to:

Surface potential driven water harvesting from fog

*Daniel P. Ura[1], Joanna Knapczyk-Korczak[1], Piotr K. Szewczyk[1], Ewa A. Sroczyk[1], Tommaso Busolo[2], Mateusz M. Marzec[3], Andrzej Bernasik[3,4], Sohini Kar-Narayan[2] and Urszula Stachewicz[1]**

[1]Faculty of Metals Engineering and Industrial Computer Science, AGH University of Science and Technology, 30-059 Kraków, Poland

[2]Department of Materials Science and Metallurgy, University of Cambridge, CB3 0FS Cambridge, United Kingdom.

[3]Academic Centre for Materials and Nanotechnology, AGH University of Science and Technology, 30-059 Kraków, Poland

[4]Faculty of Physics and Applied Computer Science, AGH University of Science and Technology, 30-059 Kraków, Poland

In this supporting information we provide histograms of PC fiber diameter distribution (Figure S1), DSC heating scans and FT-IR spectra (Figure S2), equation used for determination of crystallinity of PC samples (Equation S1), crystallinity and heat of melting for PC film and electrospun fibers (Table S1), stress-strain curves of PC samples (Figure S3), AFM topography of PC fibers and KPFM results for PC film (Figure S4), titration curve of the PC film (Figure S5), image of water droplets deposited on a PC film (Figure S6), images of cross-section thickness of PC fibers and film (Figure S7), and example mesh of model used for numerical (Figure S8).

List of abbreviations:

SYMBOL	NAME	UNIT
D_F	Fiber diameter	[µm]
x_c	Crystallinity	[%]
ΔH_f	Heat of fusion determined from DSC curve	[J·g^{-1}]
ΔH_f^0	heat of fusion for 100% crystalline PC	[J·g^{-1}]
D_{W1}	Diameter of small droplet water	[µm]
D_{W2}	Diameter of big droplet water	[µm]
D	Distance between droplet and fiber	[µm]
E	Electrical potential	[mV]

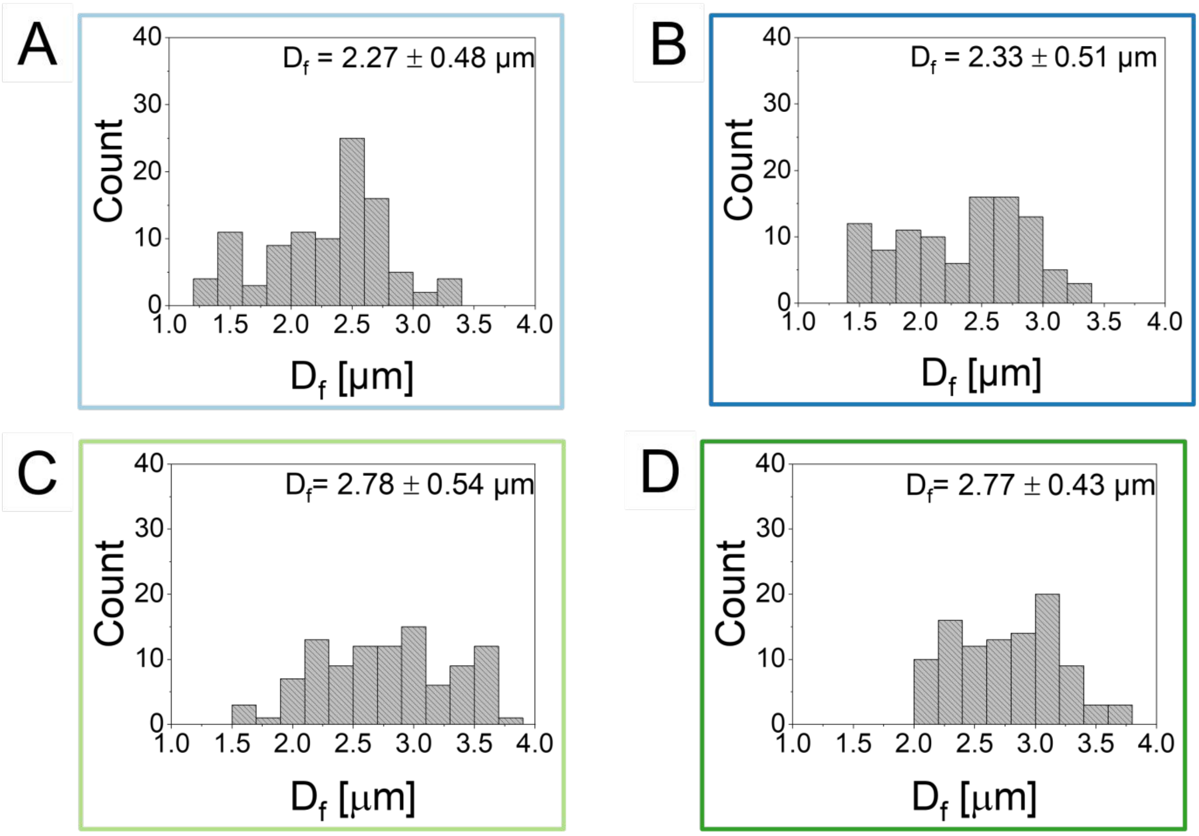

Figure S1. Histograms of fiber diameter distribution for A) PC25+, B) PC25-, C) PC40+, D) PC40-.

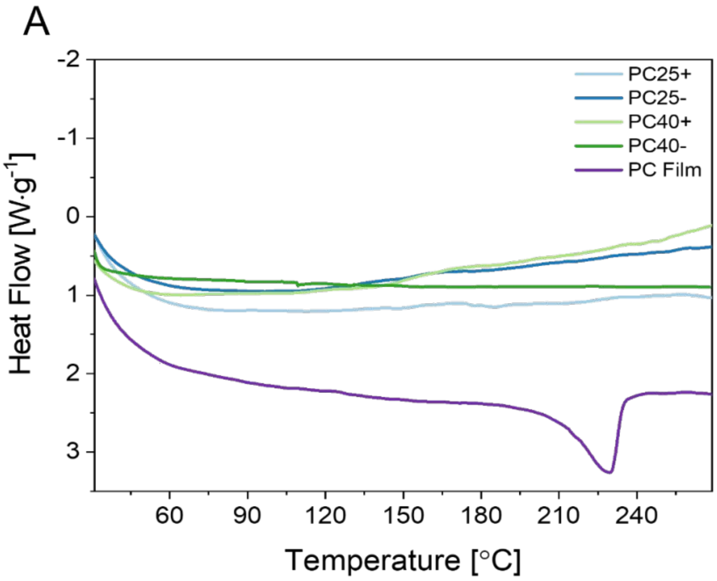

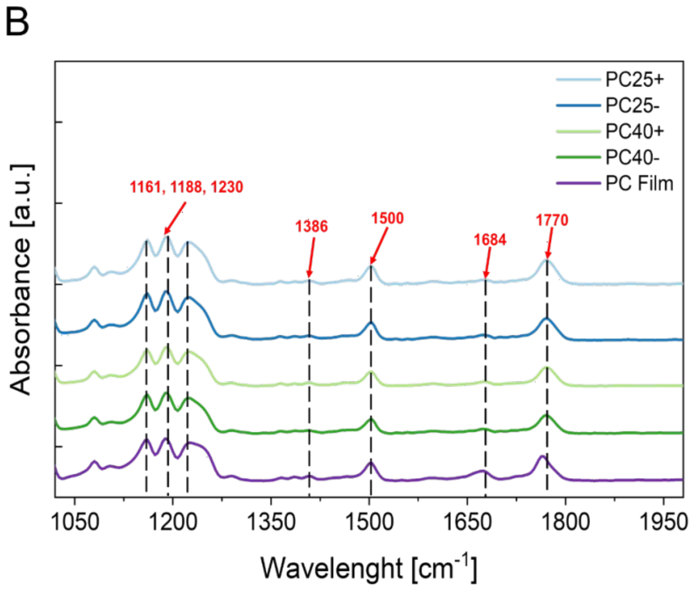

Figure S2. Characterization of electrospun PC fibers and film: A) DSC 1st heating scans, and B) FT-IR spectra with marked specific wavelength (red arrows).

The melting heat, ΔH_f, measured from the area of the melting peak (Origin software 2019b, OrginLab, Northampton, MA, USA), was taken for determination of crystallinity:

$$x_c = \frac{\Delta H_f}{\Delta H_f^0} * 100\%, \quad \text{(S1)}$$

,where ΔH_f^0 is the heat of fusion for 100% crystalline PC, taken as 147.99 J·g^{-1}.[1]

Table S1. Crystallinity and heat of melting (ΔH_f^0) for PC film and electrospun fibers after the first and the second heating runs.

	Crystalinity [%]	ΔH_f^0 - 1st heat [J/g]	ΔH_f^0 - 2nd heat [J/g]
PC Film	22.32	32.98	13.12
PC25	2.68	3.97	3.71
	2.34	3.46	2.99
PC40	2.86	4.23	3.60
	2.84	4.20	3.9

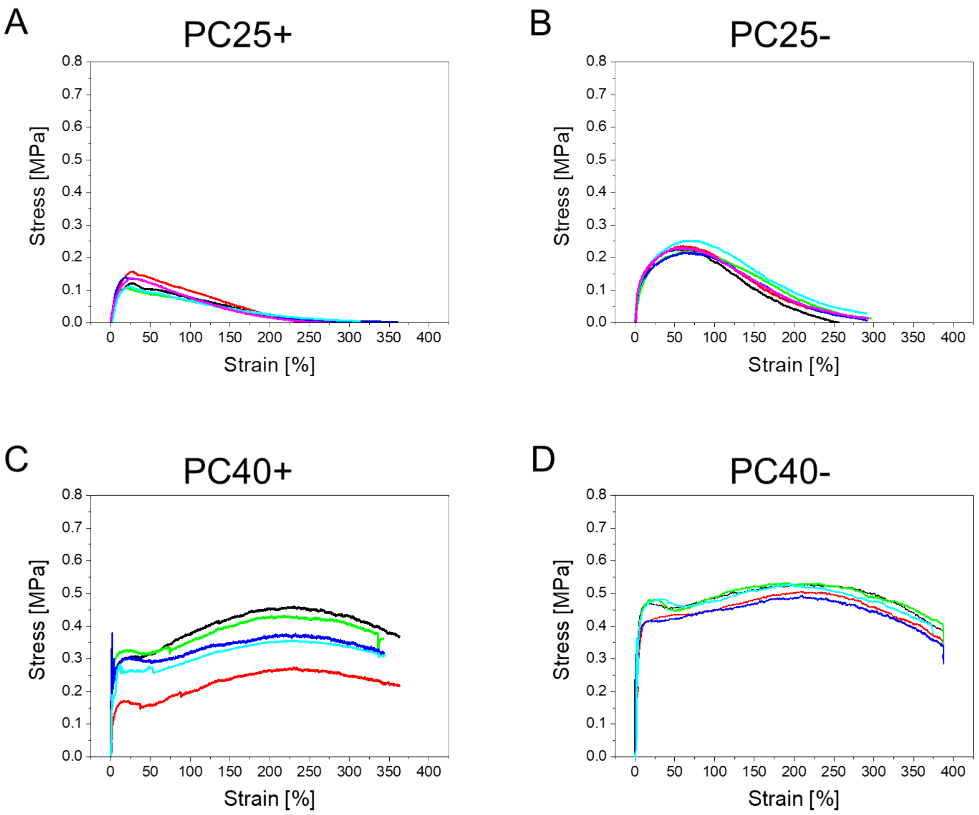

Figure S3. Stress-strain curves of A) PC25+, B) PC25-, C) PC40+, D) PC40-.

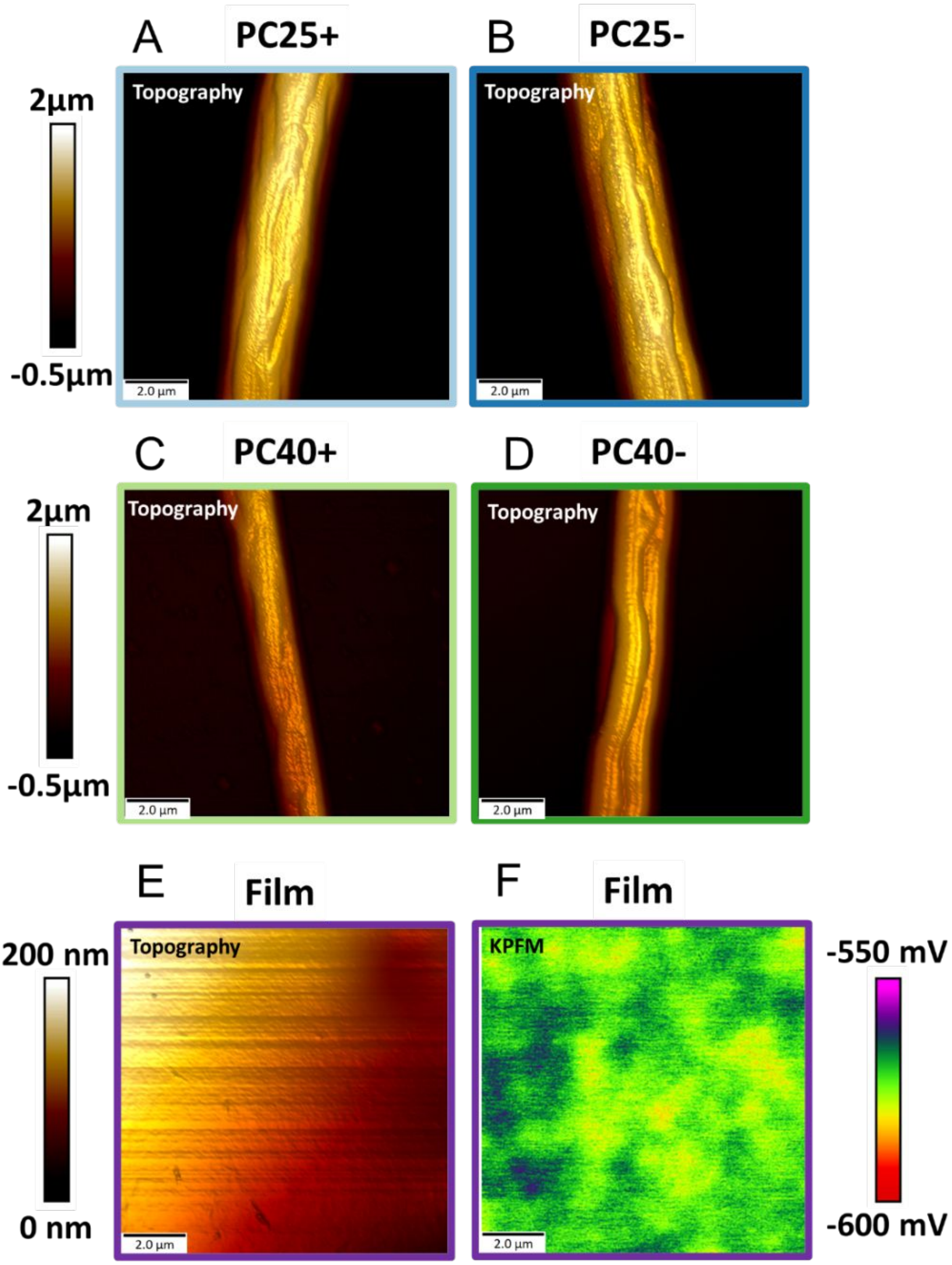

Figure S4. AFM topography of A) PC25+, B) PC25-, C) PC40+, D) PC40-, E) PC Film and KFPM results of F) PC film.

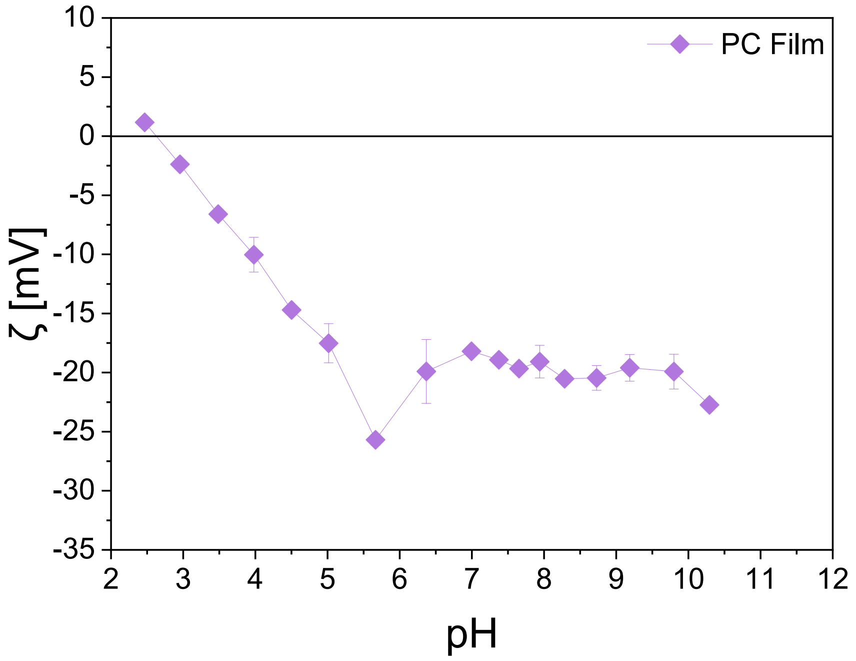

Figure S5. Titration curve for PC film in the function of pH in KCl solution indicating the zeta potential values.

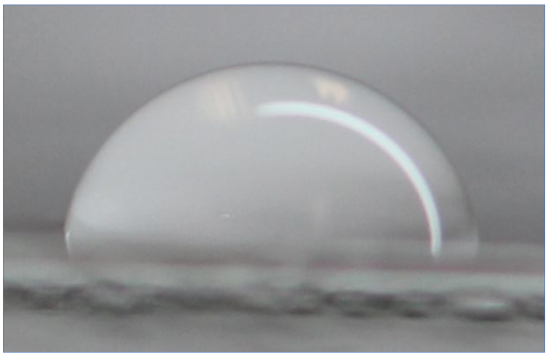

Figure S6. Representative image of water droplet deposited on PC film.

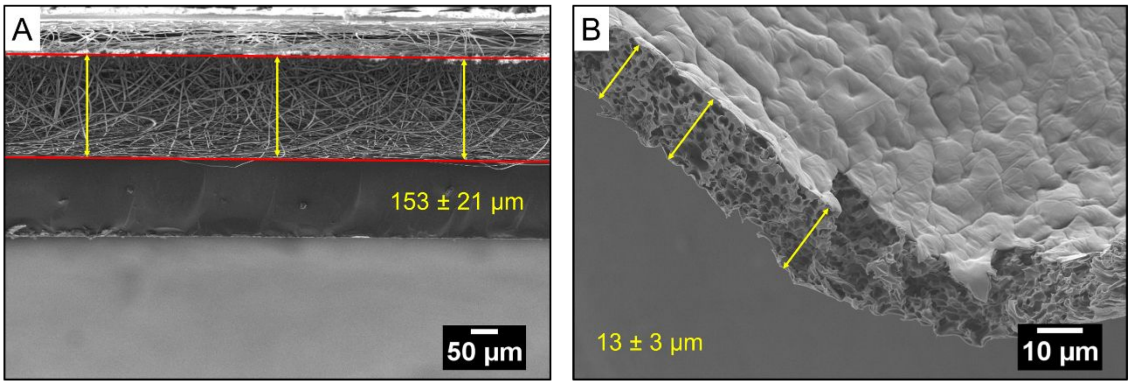

Figure S7. Representative image of cross-section thickness of PC A) fibers and B) film.

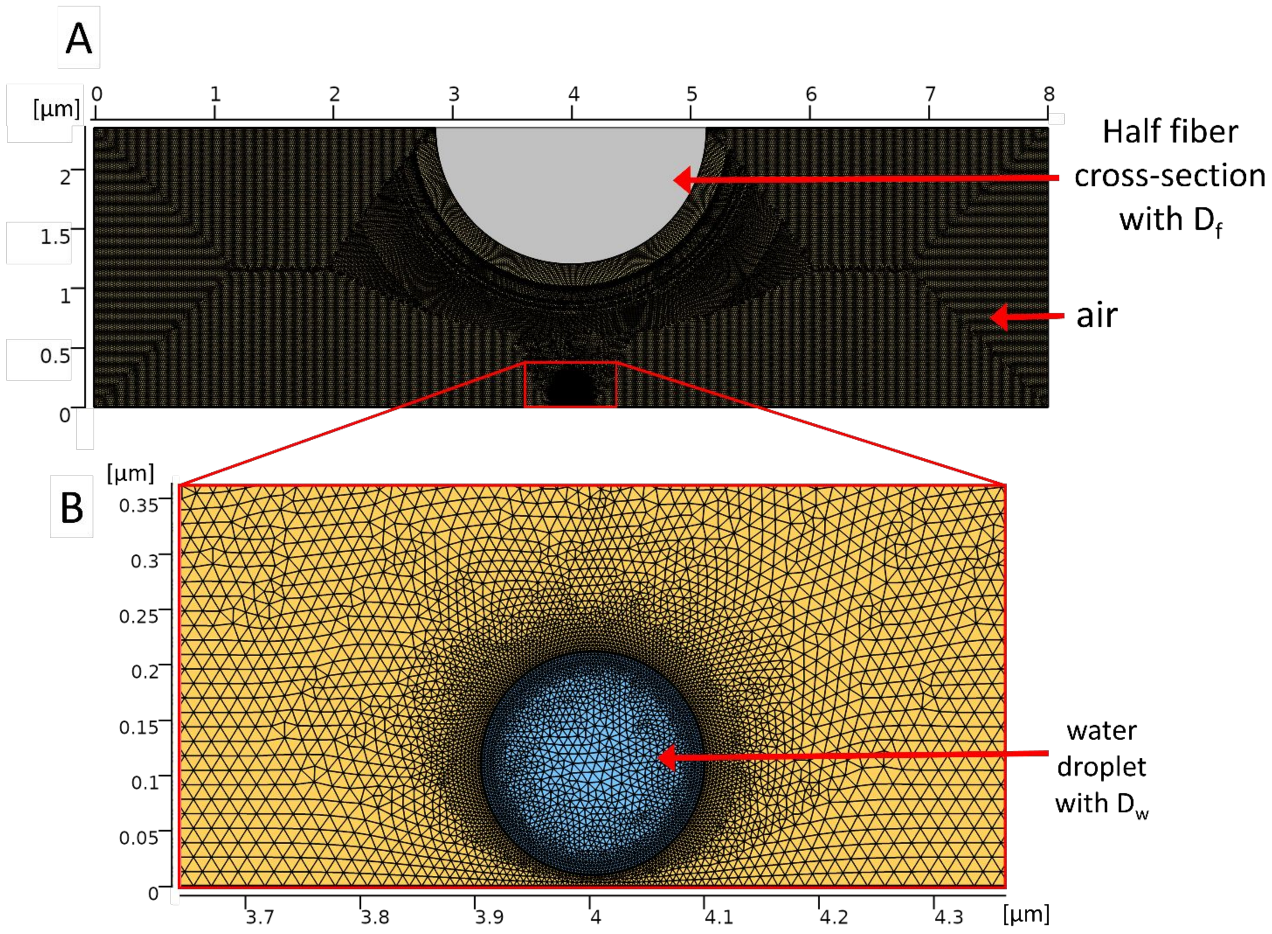

Figure S8. A) The example of full mesh and B) close-up to the mesh around the droplet used for numerical simulation of electric potential distribution between PC fiber and water droplet. D_f and D_w is diameter of fiber and water droplet, respectively.

References:

(1) Gedler, G.; Antunes, M.; Velasco, J. I. Graphene-Induced Crystallinity of Bisphenol A Polycarbonate in the Presence of Supercritical Carbon Dioxide. *Polymer (Guildf).* **2013**, *54* (23), 6389–6398. https://doi.org/10.1016/j.polymer.2013.09.050.
